# Is There a Nonperfusion Threshold on OCT Angiography Associated With New Vessels Detected on Ultra-Wide-Field Imaging in Diabetic Retinopathy?

**DOI:** 10.1167/tvst.12.9.15

**Published:** 2023-09-22

**Authors:** Hugo Le Boité, Alain Gaudric, Ali Erginay, Ramin Tadayoni, Aude Couturier

**Affiliations:** 1 Universite Paris Cité, Paris, France; 2 Ophthalmology Department, AP-HP, Hôpital Lariboisière, Paris, France; 3 Ophthalmology Department, Hôpital Fondation Adolphe de Rothschild, Paris, France

**Keywords:** OCT angiography, diabetic retinopathy, nonperfusion, new vessels

## Abstract

**Purpose:**

To determine whether the nonperfusion index (NPI) measured on widefield (WF) optical coherence tomography angiography (OCTA) could be used as an alternative method for the diagnosis of proliferative diabetic retinopathy (PDR) and to study the relationship between the NPI and the location of new vessels (NV) in eyes with PDR.

**Methods:**

Fifty-one treatment-naïve eyes with either severe nonproliferative DR (NPDR) or PDR were imaged using ultra-wide-field imaging and wide-field OCTA.

**Results:**

The NPI was significantly higher in eyes with PDR (18.94% vs. 7.51%; *P* < 0.01). Using the NPI on the whole image to assess PDR status, the area under the curve was 0.770, but the area under the curve increased when the NPI of the most peripheral circle was used (area under the curve of 0.792). Four eyes with PDR (17%) had NV outside the OCTA image field, and their mean NPI (6.15 %) did not differ from that measured in severe NPDR eyes (7.51%; *P* = 0.67) and was lower than in other eyes with PDR (21.49%; *P* = 0.023). The presence of NV in a sector was associated with a higher NPI in the same sector (29.2% vs. 6.0%; *P* < 10^−15^).

**Conclusions:**

Although the NPI was significantly higher in eyes with PDR compared with severe NPDR eyes, its measurement on the whole wide-field OCTA image was not sensitive enough to replace the detection of NV for the diagnosis of PDR.

**Translational Relevance:**

Because the presence of new vessels was related to the local nonperfusion index in the same sector, the assessment of nonperfusion outside the optical coherence tomography angiography field is important in diabetic retinopathy.

## Introduction

Screening for diabetic retinopathy (DR) is crucial to identify eyes at risk of proliferative DR (PDR), which remains a leading cause of blindness worldwide in working age adults.^1^^,^^2^ DR screening and severity score assessment are currently based on fundus examination or color fundus photography (CFP) to detect clinical signs of DR such as microaneurysms, retinal hemorrhages, microvascular changes, new vessels on the disc (NVD) and NVs elsewhere (NVE). Ultra-wide-field (UWF) CFP allows detecting DR lesions in the far periphery, and the presence of NVE outside the Early Treatment of Diabetic Retinopathy seven-field has been reported in approximately 15% of eyes with PDR.^3^^,^^4^ However, the occurrence and extent of nonperfusion areas (NPAs) secondary to capillary occlusions, which are the key factors for NV development, are not detected on fundus examination, and fluorescein angiography (FA) or optical coherence tomography angiography (OCTA) is required for proper evaluation. It has been largely demonstrated that, compared with FA, OCTA allows detecting noninvasively NVs with good sensitivity and specificity.^3^^,^^5^^–^^7^ In addition, OCTA allows visualizing the capillary network and better assessing NPAs compared with FA.^8^

Thus, using 3 mm × 3 mm and 6 mm × 6 mm macular OCTA scans, several studies have reported a decrease in retinal capillary density in DR and its correlation with DR severity.^3^^,^^9^^–^^13^ Other metrics, such as the intercapillary area,^14^ vessel tortuosity,^15^ and fractal dimension,^16^ are also impaired in eyes with DR. Nonetheless, the overlap between eyes with nonproliferative DR (NPDR) and eyes with PDR for these metrics does not allow making a reliable diagnosis of PDR, and analyzing only the macular area might not allow detecting peripheral NVE. Thus, several recent studies have used wide-field OCTA scans of 12 mm × 12 mm and 15 mm × 15 mm to accurately measure NPAs, the nonperfusion index (NPI, i.e., the NPA percentage) and to detect NVs.^5^^,^^7^^,^^17^^,^^18^ However, these studies have not identified a NPI threshold above which NVs are likely to develop, and the existence of such a threshold is still debated.^19^ The use of a single quantitative parameter such as the NPI measurement on WF-OCTA could improve the diagnosis of PDR stage in clinical practice. Therefore, the aim of this study was to determine whether the NPI measured on WF-OCTA could be used as an alternative method for the diagnosis of PDR and to study the relationship between the NPI and the location of NVs in eyes with PDR.

## Methods

### Participants

An observational case series was conducted in a tertiary ophthalmologic center in Lariboisière hospital, Paris, France. Treatment-naïve eyes with severe NPDR or PDR (simplified Early Treatment of Diabetic Retinopathy DR severity scale of the American Academy of Ophthalmology), but without macular edema, were consecutively included over a 6-month period (May to November 2021). Inclusion criteria were patients >18 years of age with type 1 or 2 diabetes and severe NPDR or PDR according to the DR severity scale of the American Academy of Ophthalmology. The criterion for the diagnosis of PDR was the presence of NVs detected on multimodal imaging: the presence of NVs was first assessed on UWF-CFP and/or by the presence of a leakage in the area of the lesion on UWF-FA, if available, determined by two retina specialists (H.L. and A.E.). It was subsequently confirmed by the presence of a flow signal in the vascular lesion and its location at the retinal surface on WF-OCTA (using the full slab, the vitreoretinal interface slab, and the B-scan with flow overlay).^20^^,^^21^ Exclusion criteria were the presence of any other retinal disorder (including high myopia) or media opacities such as vitreous hemorrhage or cataract; a history of retinal laser (macular or panretinal) or intravitreal therapy; a history of recent cataract surgery (<4 months) or pars plana vitrectomy; a poor image quality (signal strength index of <6/10) and/or presence of significant movement or shadow artifact.

Data collected included baseline demographics (gender, type of diabetes, mean diabetes duration, hemoglobin A1c level, and systemic treatments) and current ophthalmologic examination findings (the best-corrected visual acuity, slit-lamp biomicroscopy, and fundus examination). This study was conducted in accordance with the tenets of the Declaration of Helsinki. All study-related data acquisitions were approved by a French institutional review board (IRB 00008855 Société Française d'Ophtalmologie).

### Imaging Protocol

All eyes were imaged using UWF-CFP (Optos California; Optos, PLC, Dunfermline, Scotland; equipped with Optos V2 Vantage review Software) and spectral-domain OCT (Cirrus 5000 SD-OCT; Carl Zeiss Meditec Inc, Dublin, CA). In cases with uncertain NVs, UWF-FA (Optos) was performed. In addition, NPAs were assessed using OCTA (PlexElite, Carl Zeiss Meditec Inc) on a montage of five images of 12 mm ×12 mm (using the photomontage module of the device; theoretical image field of 24 mm × 24 mm).

### Image Analysis

After exporting the OCTA full-retina slabs, ImageJ software (https://imagej.net/software/fiji/) was first used to crop out low-quality areas (area removed), because NPA delineation was difficult in these areas. The low-quality areas were defined as areas with shadow or motion artifacts or vignetting that precluded NPA delineation.

Then, NPAs were automatically segmented using a python script (https://www.python.org/). After normalization, the spatial variance of the images was calculated, and NPAs were defined as areas of low variance and low intensity with a surface of >250 pixels. Then, a retina specialist corrected the NPA segmentation. Finally, we calculated the NPI as the NPA divided by the analyzable area, as shown in the formula below.

$$\begin{array}{c} Percentage_{non-perfusion}=\frac{Area_{non-perfusion}}{Area_{total}-Area_{removed}}. \end{array}$$

**Figure 1.**
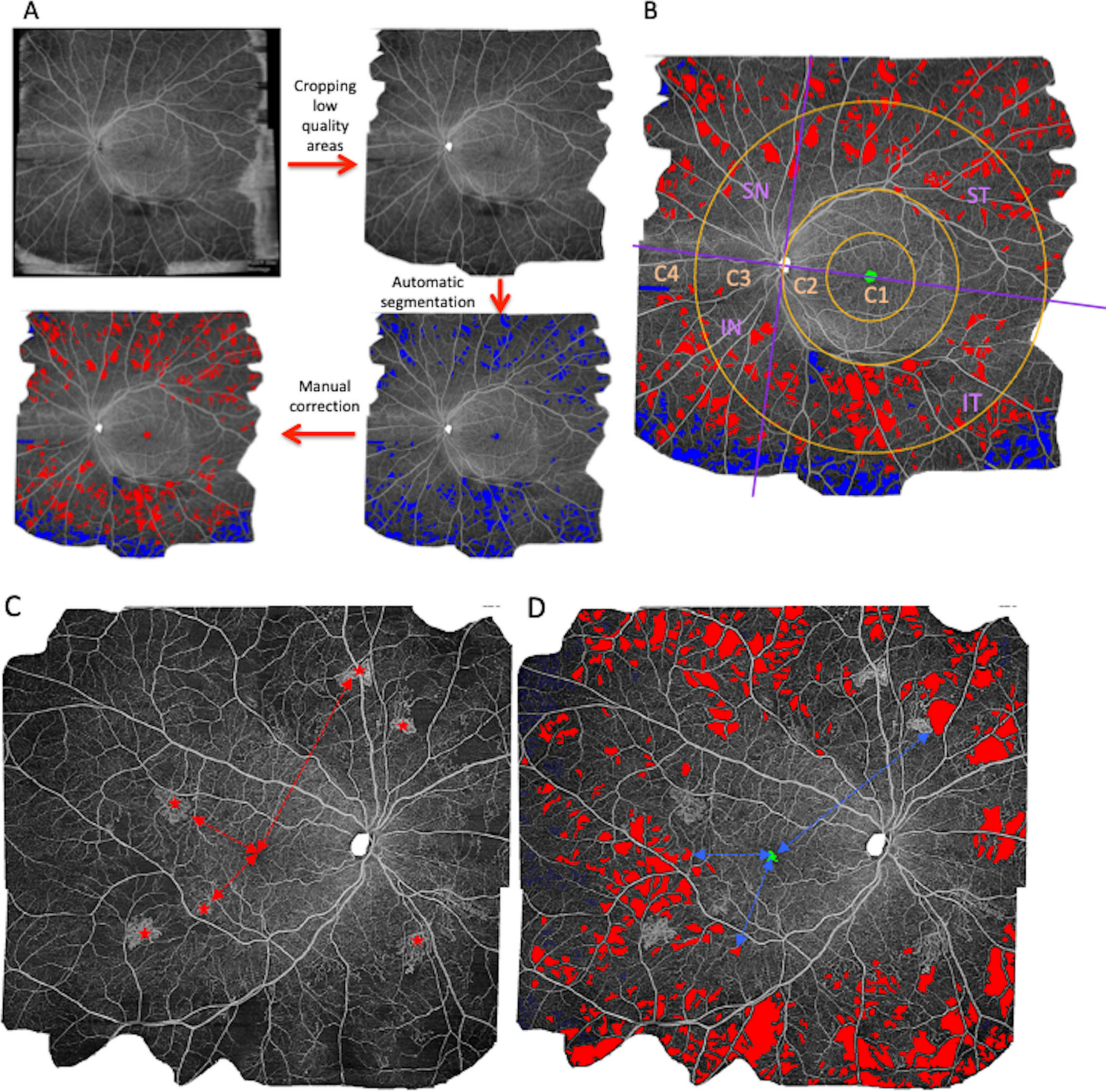
Processing of the OCTA image (5 images of 12 mm × 12 mm) to measure NPAs and calculate the NPI (percentage), on the whole image, in four retinal quadrants and in three concentric circles; and method for analyzing the relationship between the presence of NVs and the NPAs. (**A**) Method for assessing the nonperfusion: the raw image was loaded into ImageJ to crop out low-quality areas. Then, the NPAs were automatically segmented using a python script. Finally, all images were manually corrected before calculating the final NPI. (**B**) The OCTA image was divided into four quadrants (from the optic disc and fovea) and into three concentric circles from the fovea: the first circle had a radius of one-half the optic disc–fovea distance (DFD), the second circle had a radius of one DFD, and the third circle had a radius of two DFD. (**C**) OCTA image of an eye with PDR, with multiple NVs (*red stars*). Distances from the NVs to the fovea were measured and are represented by red dashed arrows. (**D**) Same image after NPA segmentation (*red surfaces*). The distances from the NPA pixels to the fovea were measured and are represented by blue dashed arrows. C1: first circle, centered on the fovea with a radius of half the DFD; C2: second circle minus first circle, centered on the fovea with a radius of 1 DFD; C3: third circle minus second circle, centered on the fovea with a radius of 2 DFD; C4: fourth circle, corresponding with the rest of the image (whole image minus third circle).

The processing workflow is shown in Figure 1A. To analyze NPA location, the OCTA montage was first divided into four quadrants (inferonasal [IN], superonasal [SN], inferotemporal [IT], and superotemporal [ST]), separated using a horizontal line passing through the optic disc and the fovea and a vertical line passing through the optic disc perpendicular to the horizontal line. Additionally, the NPI was analyzed in three concentric circles centered on the fovea: the first circle had a radius of one-half the optic disc–fovea distance, the second, a radius of 1 disc–fovea distance, and the third, a radius of 2 disc–fovea distance. The areas used for the analysis were circle 1 (C1), circle 2 minus circle 1 (C2), circle 3 minus circle 2 (C3), and the most peripheral sector outside C3 (C4). Finally, quadrants and circles were combined to create 12 retinal sectors. The superotemporal quadrant was for example divided into ST4 (intersection between the superotemporal quadrant and the zone outside the third circle), ST3, ST2, and ST1. Therefore, with 4 quadrants, 4 circles, and 12 sectors, a total of 20 zones were defined for each image (Fig. 1B).

### NPI Analysis

The mean NPI on the whole OCTA montage image, and then in each retinal sector defined above (quadrants, circles, sectors) was compared between eyes with severe NPDR and with PDR. To identify a potential NPI threshold that could be associated with the presence of NVs, a diagnostic performance analysis was performed using the NPI (whole image, quadrants, circles, or sectors) as a diagnostic variable to detect the PDR status. The NPIs in sectors with or without NVs in eyes with PDR were also compared. Finally, the distance between each NV site and the fovea, and the distance between each NPA and the fovea were measured (Figs. 1C and D). The correlation between the minimum and mean distances from the NPA to the fovea and the overall NPI, as well as between the shortest distance from NV to the fovea and the overall NPI were calculated.

### Statistics

All statistical analyses were performed using R software (https://www.r-project.org/). Means were compared using the Student *t*-test (2 groups) or an analysis of variance (>2 groups), if the group size was sufficient, a Wilcoxon test (2 groups) or a Kruskal–Wallis test (>2 groups) was used otherwise. A correlation analysis was performed with a Pearson coefficient if at least one of the variables had a normal distribution, or with a Spearman coefficient otherwise. For the diagnostic performance analysis, the receiver operating characteristic curve was plotted, and the area under the curve (AUC), optimal cutoff (Youden index), sensitivity, specificity, positive and negative predictive values were calculated.

## Results

A total of 51 eyes of 30 patients were included (mean age, 49.67 ± 14.12 years; 70% of men): 24 eyes had PDR and 27 had severe NPDR. In all the 24 eyes with PDR, NVs were detected on UWF-CFP. Additionally, UWF-FA was performed in 19 eyes, including 7 eyes with PDR, and confirmed the presence of NVs in these 7 eyes, whereas severe NPDR was diagnosed in 12 eyes and ruled out the presence of NVs. WF-OCTA allowed detecting the presence of NVs in 20 of 24 eyes with PDR.

Indeed, four eyes (7.8%) had peripheral NVs detected only on UWF-CFP; they were not visible on WF-OCTA. Conversely no NV was detected only on WF-OCTA. Nineteen patients (63%) had type 2 diabetes, and the mean hemoglobin A1c level was 8.79 ± 1.96% (range, 5.0%–12.3%). Seventeen patients (71%) received antihypertensive treatment. Forty-two eyes (82%) were phakic. The mean best-corrected visual acuity was 0.11 ± 0.17 logMAR (range, 0–0.8 logMAR).

### NPI and Nonperfusion Distribution

**Figure 2.**
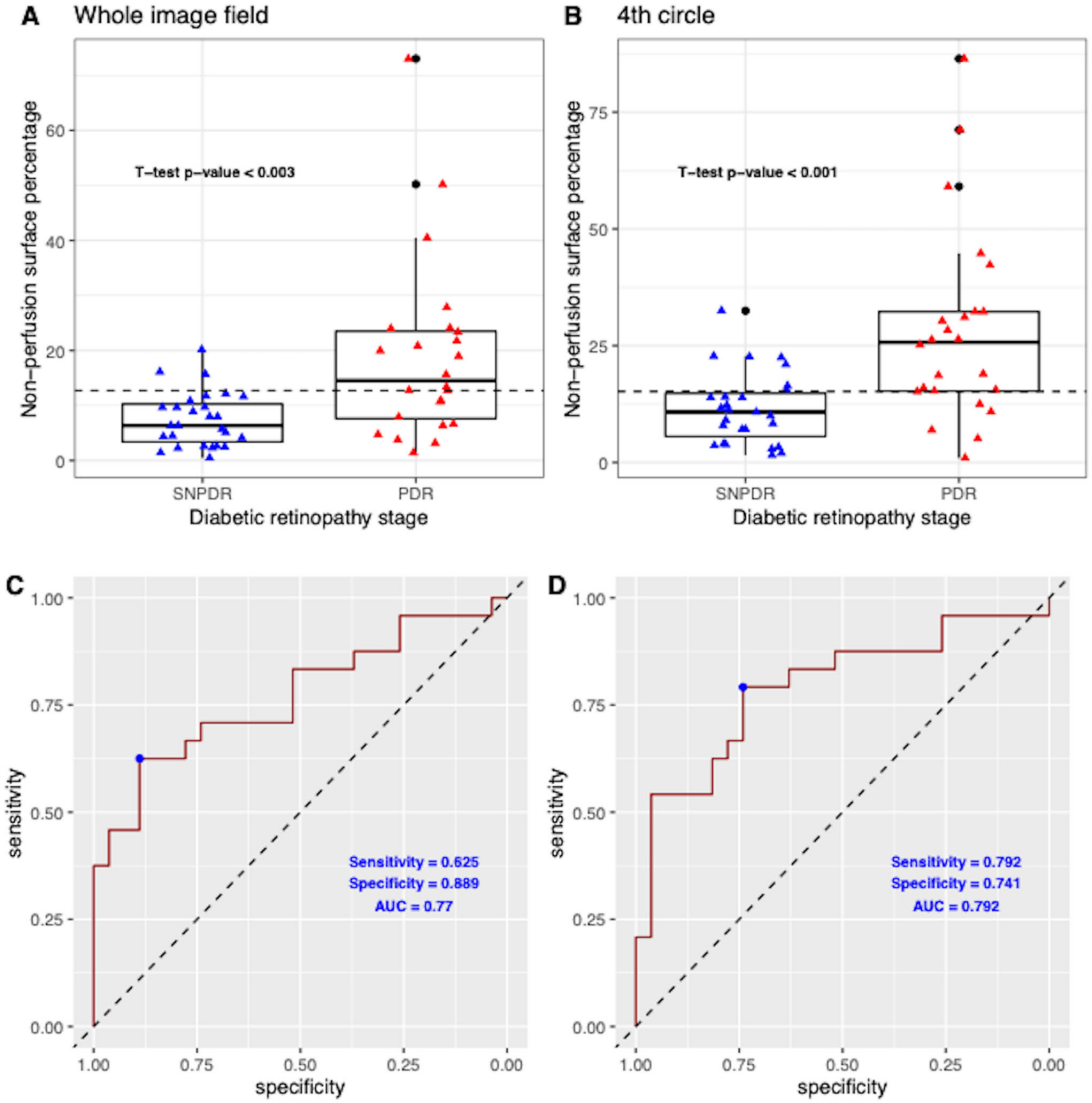
Diagnostic performance for PDR using the measured NPI (NPA percentage). (**A** and **B**) NPI distributions in eyes with severe NPDR (SNPDR) and eyes with PDR on the whole image (**A**), and in the most peripheral sector of the OCTA image (fourth circle). The dashed lines correspond to the optimal NPI cutoffs (Youden index) of 12.69% and 15.22%, respectively. The mean NPI was significantly higher in eyes with PDR than in severe NPDR eyes: 18.94 ± 16.42% (range, 1.41%–73.03%) versus 7.51 ± 4.97% (range, 0.46%–20.15%), respectively (*P* < 0.01). (**C** and **D**) Receiver operating characteristic (ROC) curves using the NPI as a diagnostic tool to detect PDR on the whole image and in the fourth circle sector, respectively. Using the NPI measured on the whole image, the sensitivity was limited to 0.625, with a specificity of 0.889 and an AUC of 0.770. However, when using the NPI measured in the fourth circle, the sensitivity increased to 0.792, with a specificity of 0.741 and an AUC of 0.792.

The mean NPI on the whole WF-OCTA image was 12.89 ± 13.04% (range, 0.46%–73.03%) and was higher in eyes with PDR than in severe NPDR eyes: 18.94 ± 16.42% (range, 1.41%–73.03%) versus 7.51 ± 4.97% (range, 0.46%–20.15%), respectively (*P* < 0.01). The respective distributions are shown in Figure 2A. The mean overall area removed for the NPI calculation was 16.33 ± 6.21%, mainly owing to the vignetting effect of the montage, and did not differ between the severe NPDR and PDR groups (14.92 ± 6.54% and 17.91 ± 5.67%, respectively; *P* = 0.09). Moreover, the area removed did not correlate with the NPI (rho = 0.03; *P* = 0.84).

Among demographics and clinical parameters, only the gender was significantly associated with nonperfusion: the NPI was higher in women compared with men (16.92 ± 12.49% vs. 10.87 ± 13.02%, respectively; *P* = 0.018), probably owing to an uneven distribution (65% of women in the PDR group compared with 38% of men). The best-corrected visual acuity positively correlated with the NPI, but without reaching significance (Spearman coefficient = 0.281; *P* = 0.051). Other parameters were not significantly associated with the NPI, as shown in Supplementary Table S1.

**Figure 3.**
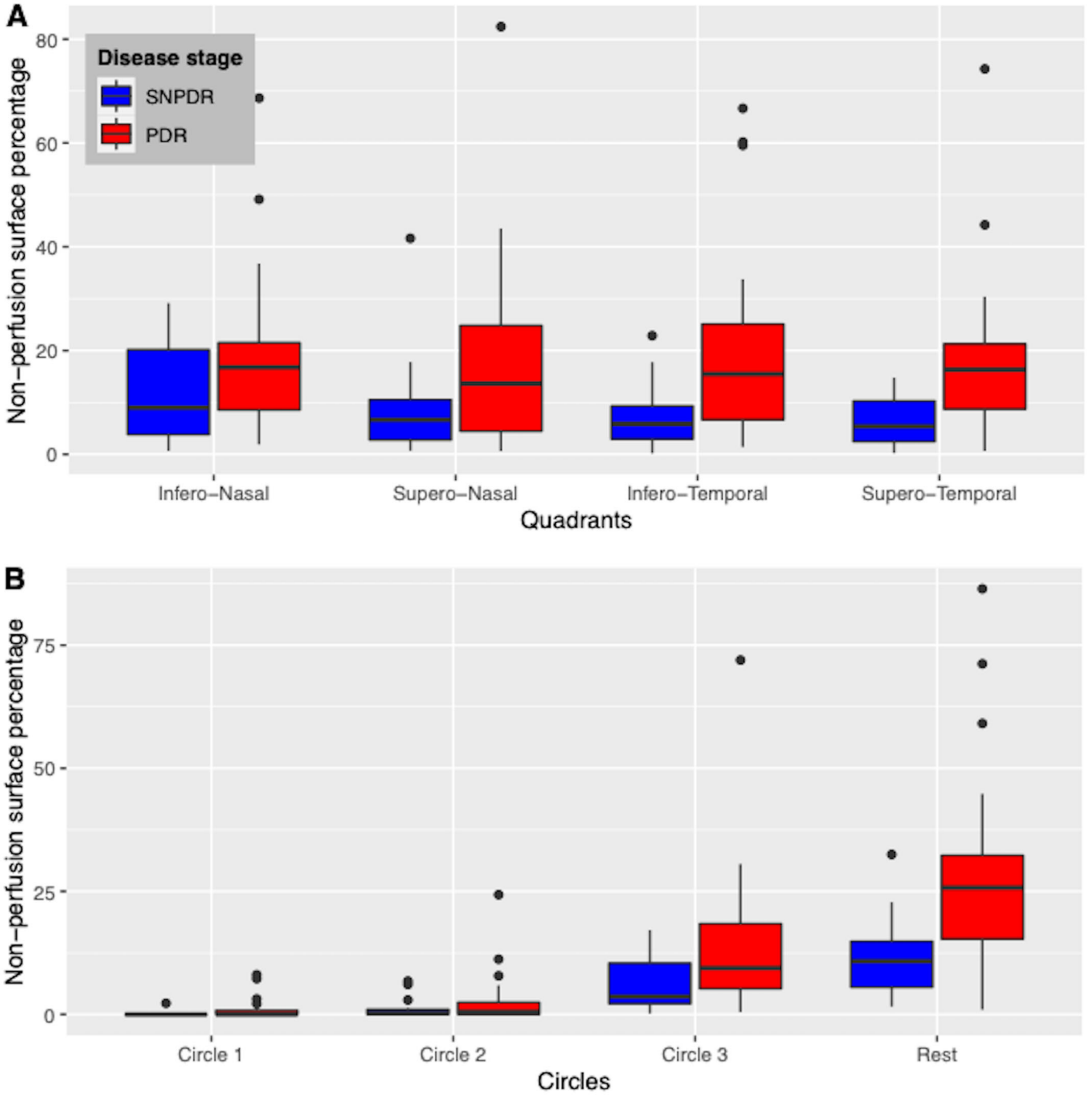
Distribution of the NPI (NPA percentage) in the retinal sectors (quadrants and circles) of the OCTA image field. (**A**) NPI distribution in the 4 quadrants in eyes with SNPDR (*blue boxplots*) and in eyes with PDR (*red boxplots*). The mean NPI did not significantly differ between the 4 retinal quadrants (analysis of variance [ANOVA] test; *P* = 0.71), but in each quadrant, the mean NPI was significantly higher in the PDR group compared with the severe NPDR group (detailed results in Table) (**B**) NPI distribution in the four retinal circles in eyes with severe NPDR (*blue boxplots*) and in eyes with PDR (*red boxplots*). The mean NPI was significantly higher in C3 and C4 compared with C1 and C2 (ANOVA test, *P* <10^−10^), and the mean NPI in C3 and C4 was significantly higher in eyes with PDR compared with eyes with severe NPDR (detailed results in Table).

The mean NPI did not significantly differ between the 4 retinal quadrants (analysis of variance, *P* = 0.71) (Fig. 3A and Table). Regarding nonperfusion location in the circles centered on the fovea, the mean NPI was significantly higher in C3 and C4 compared with C1 and C2 (analysis of variance; *P* < 10^−10^) (Fig. 3B and Table). The NPI in each circle correlated with the NPI on the whole image, with a stronger correlation for C3 and C4 (correlation coefficients of 0.61, 0.55, 0.89, and 0.97 for C1, C2, C3, and C4 respectively; *P* < 10^−4^).

**Table.** NPI Distribution in Different Sectors of the OCTA Image

	Quadrants						Circles					
	Whole Image	SN	IN	ST	IT	ANOVA	Whole Image	C1	C2	C3	C4	ANOVA
Overall	12.89 ± 13.04 (0.46–73.03)	12.89 ±14.33 (0.6–82.43)	14.86 ± 12.95 (0.66–68.67)	11.71 ± 12.66 (0.22–74.28)	13.08 ± 14.75 (0.18–66.68)	0.71	12.89 ± 13.04 (0.46–73.03)	0.52 ± 1.6 (0–8.05)	1.77 ± 3.97 (0–24.32)	9.81 ± 11.6 (0.18–71.96)	19.3 ± 17.2 (0.97–86.44)	<10^−10^
SNPDR	7.51 ± 4.97 (0.46–20.15)	8.46 ± 8.29 (0.7–41.64)	10.77 ±8.36 (0.66–29.13)	6.21 ± 4.28 (0.22–14.77)	6.91 ± 5.57 (0.18–22.85)	0.08	7.51 ± 4.97 (0.46–20.15)	0.08 ± 0.44 (0–2.27)	0.93 ± 1.71 (0–6.77)	6.01 ± 5.07 (0.18–17.12)	11.57 ± 7.69 (1.6–32.49)	<10^−10^
PDR	18.94 ± 16.42 (1.41–73.03)	17.88 ± 17.88 (0.6–82.43)	19.46 ± 15.62 (1.9–68.67)	17.9 ± 15.89 (0.63–74.28)	20.01 ± 18.53 (1.43–66.68)	0.96	18.94 ± 16.42 (1.41–73.03)	1.01 ± 2.21 (0–8.05)	2.71 ± 5.39 (0–24.32)	14.09 ± 15.09 (0.48–71.96)	28.01 ± 20.66 (0.97–86.44)	<10^−10^
Student *t*-test	0.003	0.024	0.02	0.002	0.003	–	0.003	0.053	0.132	0.019	0.001	–
Rho	1	0.87	0.78	0.97	0.94	–	1	0.61	0.55	0.89	0.97	–

Mean NPI (NPA percentage) on the whole image, in the four quadrants and in the four concentric circles, in the whole cohort, in eyes with SNPDR and in eyes with PDR. The mean NPI did not differ between the different quadrants. The NPI was significantly higher in eyes with PDR than in eyes with SNPDR on the whole image as well as in each quadrant, and in the two most peripheral concentric circles (C3 and C4).

All results are shown as a mean (percentage) ± standard deviation (range). The Student *t*-test was used to compare the severe NPDR and PDR groups. Rho: correlation coefficient calculated between the NPI on the whole image and the local NPI in different quadrants and circles. All tests were statistically significant, with *P* values of <10^−10^ (for the quadrants) and <10^−4^ (for the circles). Analysis of variance (ANOVA) was calculated between the NPI distributions in the different quadrants.

C1, first circle, centered on the fovea with a radius of half the optic disc-fovea distance (DFD); C2, second circle minus first circle, centered on the fovea with a radius of 1 DFD; C3, third circle minus second circle, centered on the fovea with a radius of 2 DFD; C4, fourth circle, corresponding to the rest of the image (whole image minus third circle).

### NPI Threshold and Presence of Neovascularization

Using the NPI on the whole image to determine the PDR status, the diagnostic performance AUC was 0.770 (optimal NPI cutoff, 12.69%; sensitivity, 0.625; specificity, 0.889; positive predictive value, 0.824; negative predictive value, 0.706) (Fig. 2C). However, when the NPI in the different retinal sectors (quadrants, circles, sectors) was used, PDR diagnostic performances changed: the highest performances were found in the most peripheral circle (C4, AUC = 0.792; sensitivity = 0.792; specificity = 0.741; optimal cutoff = 15.22%) (Fig. 2D), in the superotemporal quadrant (AUC = 0.784; sensitivity = 0.542; specificity = 1; optimal cutoff = 15.79%) and inferotemporal quadrant (AUC = 0.773; sensitivity = 0.667; specificity = 0.778; optimal cutoff = 10.41%). The results for each sector are detailed in Supplementary Table S2.

### Local Relationship Between Neovascularization and the NPI

In the PDR group, 4 eyes (17%) had NVE outside the OCTA image field that were detected only on UWF imaging, 16 eyes (67%) had NVE inside the image field, and 4 eyes (17%) had NVD. A significant trend toward an increase in the NPI was observed between eyes with NVE outside the image field (6.15 ± 4.91%, with 3 out of 4 cases <6.65%), eyes with NVE inside the image field (15.33 ± 7.73%), and eyes with NVD (46.13 ± 21.7%) (*P* = 0.0056) (Supplementary Fig. S1).

The highest probabilities of finding at least one NV site in a given sector were found in ST3, IT3, SN4, and IN4 (27.45%, 27.45%, 27.45%, and 23.53%, respectively). All the probabilities for the presence of a NV are shown in Supplementary Table S4.

**Figure 4.**
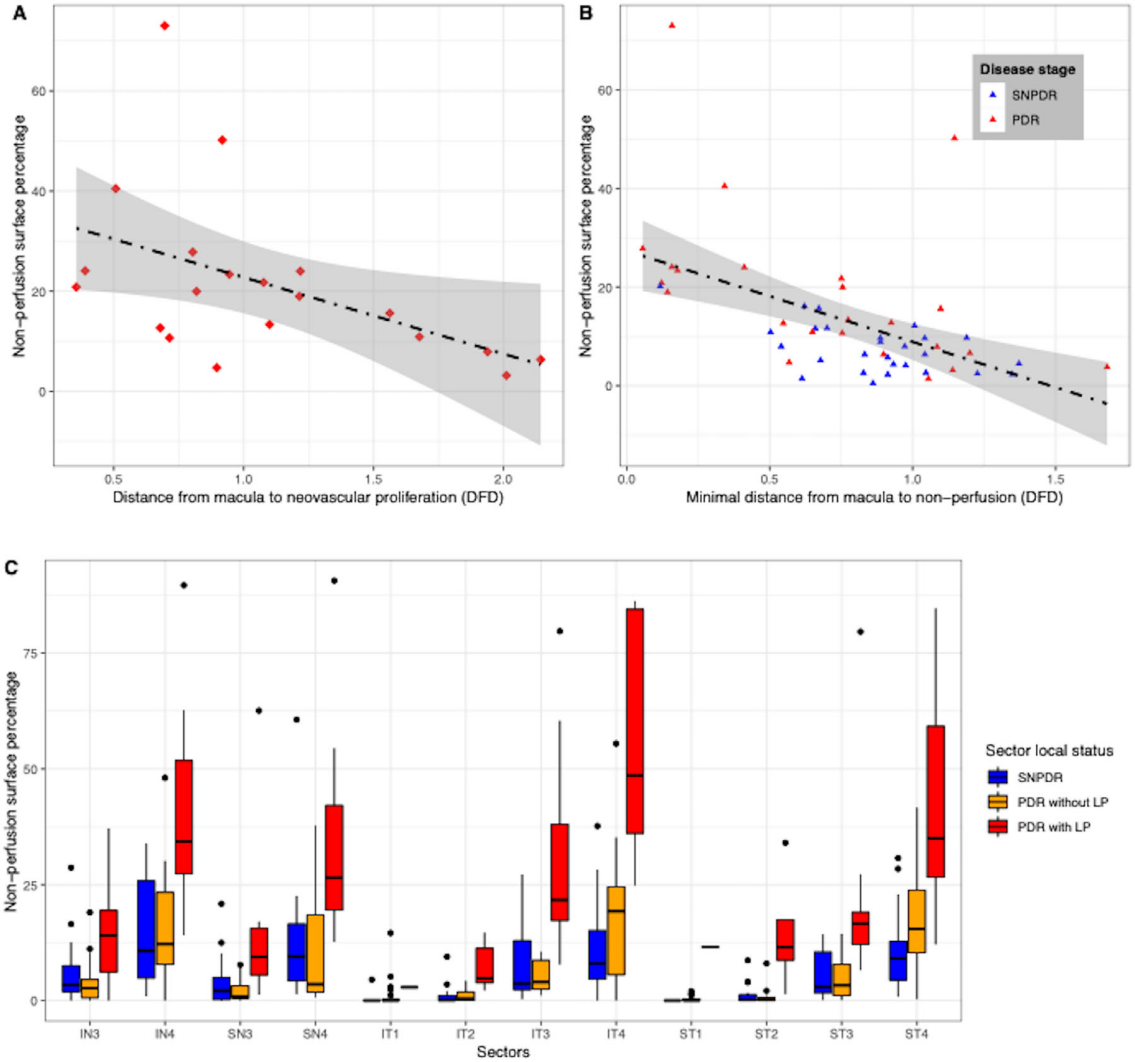
Relationship between the presence of NVs, the NPI (NPA percentage) and the location of retinal NPAs. The distance from the closest NV or the closest NPA to the fovea is expressed as a fraction of the optic disc–fovea distance (DFD). (**A**) Correlation between the distance from the closest NV to the fovea and the NPI, in eyes with PDR. Pearson correlation coefficient: rho = –0.48 (*P* = 0.031). There was a significant negative correlation, meaning that the closer the NV was to the fovea, the higher the NPI was. (**B**) In the whole cohort, correlation between the distance from the closest NPA to the fovea and the NPI. Pearson correlation coefficient: rho = –0.52 (*P* < 0.0001). There was a significant negative correlation, meaning that the closer the closest NPA was to the fovea, the higher the NPI was. (**C**) Distribution of the NPI in the 12 retinal sectors. In this plot, each point represents one individual sector in 1 eye, with its corresponding NPI. We compared sectors of eyes with SNPDR (*blue boxplots*) with sectors of eyes with PDR but without NVs in the analyzed sector (*orange boxplots*) and with sectors of eyes with PDR with local NVs in the analyzed sector (*red boxplots*). The mean NPI was significantly higher in sectors with NVs (*red boxplots*) than in sectors without NVs (blue and orange boxplots): 29.2 ± 23.2% versus 6.0 ± 9.0%, respectively (*P* < 10^−15^). The difference remained significant in all sectors analyzed individually. Interestingly, there was no difference in mean NPI in the sectors without NVs between eyes with PDR (*orange boxplots*) and eyes with severe NPDR (*blue boxplots*): 6.05 ± 10.2% and 5.95 ± 8.2% respectively (*P* = 0.91), reinforcing the hypothesis of a local relationship between NPAs and the presence of NVs. DFD, optic disc–fovea distance; LP, local proliferation (presence of NVs in a given sector). Numbers associated with quadrant abbreviation: 1 = first circle, centered on the fovea with a radius of one-half the DFD; 2 = second circle minus first circle, centered on the fovea with a radius of 1 DFD; 3 = third circle minus second circle, centered on the fovea with a radius of 2 DFD; 4 = fourth circle, corresponding to the rest of the image (whole image minus third circle). Example: IN3 corresponds with the IN quadrant intersected with the third circle.

In eyes with PDR, a negative correlation was observed between the distance from the closest NV site to the fovea and the NPI on the whole WF-OCTA image (rho = −0.48; *P* = 0.031) (Fig. 4A), meaning that the NPI was higher in eyes with a NV closer to the fovea. Similarly, in the whole cohort, a negative correlation was found between the minimum distance from the closest NPA to the fovea and the NPI (rho = −0.52; *P* < 0.0001) (Fig. 4B). In other words, the closer the NPA was to the fovea, the higher the overall NPI was.

When comparing the NPI in each sector, the mean local NPI was significantly higher in sectors with NVs than in sectors without NVs (29.2 ± 23.2% vs. 6.0 ± 9.0%, respectively; *P* < 10^−15^) (Fig. 4C). Additionally, a subsector analysis was performed, with the same comparison, but assessing one by one each of the different types of retinal sectors (i.e., only the C1 sectors of all eyes, then only the C2 sectors of all eyes, and so on). In the subsector analysis, the local NPI remained significantly higher in sectors with NVs compared with sectors without NVs. Interestingly, there was no difference in mean NPI in the sectors without NVs between the eyes with PDR and the eyes with severe NPDR (6.05 ± 10.2% and 5.95 ± 8.2%, respectively; *P* = 0.91). The local relationship between the presence of NVs and the NPI is shown in Supplementary Figure S2. When the local NPI in a retinal sector was used as a predictive variable for the presence of local NVs, the AUC was 0.882 (optimal cutoff, 11%; sensitivity, 0.82; specificity, 0.81).

## Discussion

In this study, we aimed to determine whether the NPI measured on WF-OCTA could be used as an alternative diagnostic method for PDR and to study the relationship between the NPI and the location of NVE in eyes with PDR. Our results confirmed the assumption that the NPI, even in the wider OCTA field available, was not sensitive enough to fully replace the detection of NVs in DR.

In this series, the mean NPI was higher in eyes with PDR compared with severe NPDR eyes, as previously reported using standard 12 mm × 12 mm OCTA scans.^18^^,^^22^ However, in our series, there was a significant overlap of NPI between severe NPDR and eyes with PDR. Therefore, the optimal NPI threshold identified in the receiver operating characteristic curve analysis (12.69%) showed a sensitivity of only 0.63, with an AUC of 0.77, to identify the PDR status, which would be too low for use in clinical practice. This finding could be explained by the fact that some eyes with PDR could have NPAs mainly located in the far periphery outside the OCTA field, and, therefore, the NPI measured on WF-OCTA could not reflect the overall nonperfusion distribution in these eyes.

Indeed, in our series, the subsector analysis found an uneven NPI distribution, with higher values in the peripheral sectors, which is consistent with the results of Wang et al.,^23^ who have investigated the NPI in eyes with different DR stages, imaged using the same type of OCTA montage and have found that NPAs were mainly located in the most peripheral circular sector (50°–100° of view). Recently, the NPA distribution in eyes with DR has been assessed using UWF-FA and several studies have shown that there is a NPI gradient between the far periphery and the posterior pole, with higher NPI in the periphery.^24^^–^^28^ Niki et al.^29^ have described capillary nonperfusion profiles in NPDR eyes using FA, and have already shown in 1984 that 2.6% and 61.2% of eyes had a peripheral and midperipheral nonperfusion distribution, respectively.

Given the uneven distribution of nonperfusion, when we used only the NPI in the most peripheral sector (C4) to identify PDR status, the diagnostic performance increased (sensitivity, 0.792; AUC, 0.792). Previous studies have reported the low diagnostic efficacy of individual parameters on 3 × 3-mm OCTA images to differentiate NPDR from PDR. Combining selected OCTA parameters has improved the diagnostic performances, but it may not be easy to use it in clinical practice. After combining selected 3 mm × 3 mm OCTA metrics, Ashraf et al.^30^ have achieved better performances. However, these studies have mainly included patients with mild NPDR, whereas we only included patients with severe NPDR, so that the difference in severity was greater between our two groups. In this study, we deliberately only included eyes with severe NPDR in the NPDR group, because this is the critical stage that should be detected in clinical practice to treat early and prevent vision-threatening complications.

Several hypotheses could be discussed to explain why the NPI measured on WF-OCTA did not allow accurately detection of the presence of neovascularization visible on UWF images in all eyes with PDR.

First, the field of the OCTA image was too limited to detect all NVE sites, even though NVE were mainly located in mid-peripheral sectors, as already described.^24^^–^^26^ In our series, four eyes (17%) had peripheral NVs detected only on UWF imaging and not on WF-OCTA. Russell et al.^27^ have reported a high NVE detection rate using simulated WF-OCTA, with a montage of five images, compared with UWF-FA, with only 2% of cases with NVE detected on UWF-FA outside the simulated WF-OCTA image field. However, the WF-OCTA image field is smaller in clinical practice than the simulated one, because the borders of the image are frequently of low quality.

Interestingly, we found that the NPI was lower in these eyes than in other eyes with PDR and did not significantly differ from that measured in severe NPDR eyes. This finding suggests that NVs could develop, in some patients, only in the far periphery, in response to local peripheral NPA, but with a relatively low NPI in the posterior pole. In contrast, the NPI was significantly higher in eyes with NVD (17%) than in eyes with only NVE, reinforcing the assumption that NVD could develop secondary to severe global retinal ischemia.

Our analysis also showed a local relationship between the NPA and the presence of NVE. We found a correlation between the distance from NVE to the fovea and the NPI on the whole OCTA image: eyes with NVE closer to the fovea had a higher NPI, which could suggest a form of centripetal progression of nonperfusion, or different NPA distribution profiles depending on disease severity.^29^ Similarly, the NPI correlated with the minimum NPA–fovea distance: eyes with NPA closer to the fovea had a higher NPI. This centripetal progression of nonperfusion could be due to an autoregulation of retinal perfusion to preserve the macula.^31^^,^^32^ We could also assume that the detection of NPAs on OCTA could be limited in the central region owing to the higher capillary density and the presence of three capillary plexuses compared with the midperiphery,^33^ making NPAs less visible. The disappearance of the intermediate capillary complex in the midperiphery, at a distance of approximately 6 to 7 mm from the fovea,^33^ could explain the higher detection of NPAs in the periphery.

Last, when comparing the NPI in sectors with and without local NVE, the NPI in sectors without local NVE in eyes with PDR did not differ significantly from that in sectors in NPDR eyes. In other words, in eyes with PDR, the nonperfusion was mainly located in sectors with local NVE. The diagnostic performance of the local NPI in retinal sectors was associated with an AUC of 0.882 (optimal NPI cutoff, 11%; sensitivity, 0.82; specificity, 0.81). These results highlighted a relationship between the presence of NVE and the local NPA in the same retinal sector, and not only with the overall retinal nonperfusion.

Our study has some limitations. First, we included a relatively small number of eyes. Second, the imaging modalities used for the diagnosis of PDR were UWF-CFP and WF-OCTA in all eyes, and UWF-FA in 19 eyes only. Because these image modalities have different sensitivities for the detection of peripheral NVE,^27^ this result could represent a limitation for the definition of PDR in this study. Third, we cropped out low-quality areas of the WF-OCTA montage (16.33 ± 6.21% of the 24 mm × 24-mm montage), which could have biased NPI estimation. However, the area removed did not significantly differ between the two groups and did not correlate with the NPI. Finally, considering that the acquisition of the WF-OCTA montage was time consuming and could be difficult in some patients, and that the NPA delineation needed a manual correction, the clinical applicability could be limited in practice. In the future, longitudinal studies aiming at assessing the change in nonperfusion could be a critical point in understanding NV development.

To conclude, in this series, we showed that, although the NPI measured on WF-OCTA was significantly higher in eyes with PDR compared with eyes with severe NPDR, OCTA did not allow detecting all cases of PDR diagnosed on UWF-CFP or FA. The ability of WF-OCTA to accurately differentiate eyes with PDR from eyes with severe NPDR was limited owing to the relatively low NPI measured on OCTA in eyes with NVE outside the OCTA image field and to the local relationship between NVE and the NPA in each retinal sector.

## Supplementary Material

Supplement 1
