# Supplementary material for: Is There a Nonperfusion Threshold on OCT Angiography Associated With New Vessels Detected on Ultra-Wide-Field Imaging in Diabetic Retinopathy?
Source: Transl Vis Sci Technol. 2023 Sep 22;12(9):15. doi: 10.1167/tvst.12.9.15 (PMC10519435; doi:10.1167/tvst.12.9.15)
Supplement: Supplement 1 [file tvst-12-9-15_s001.pdf]

SUPPLEMENTARY FIGURES

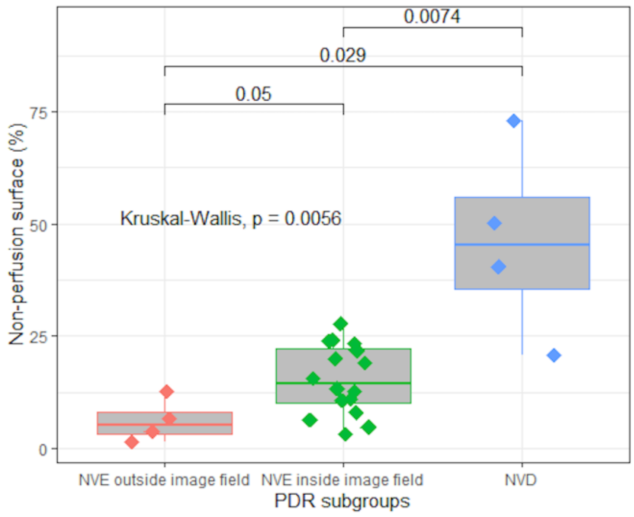

Supplementary Figure 1: In the proliferative diabetic retinopathy (PDR) group, distribution and statistical comparison of the overall non-perfusion index (NPI, non-perfusion area percentage) between eyes with new vessels elsewhere (NVE) outside the image field (red points), NVE inside the image field (green points), or new vessels in the optic disc (NVD,

blue points). We observed a significant trend towards an increase in NPI between eyes with NVE outside the image field (6.15 ± 4.91%, with 3 out of 4 cases less than 6.65%), eyes with NVE inside the image field (15.33 ± 7.73%), and eyes with NVD (46.13 ± 21.7%), using a Kruskal-Wallis test ($p = 0.0056$). The 2-by-2 comparisons of the 3 groups, using a Wilcoxon test, were all statistically significant.

Abbreviations: PDR: proliferative diabetic retinopathy; NVE: new vessels elsewhere; NVD: new vessels in the optic disc.

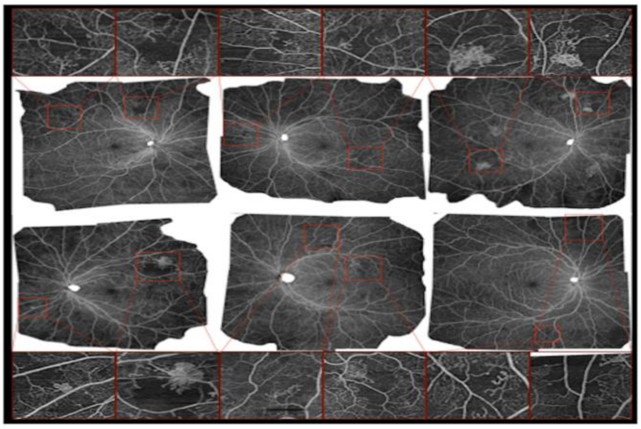

Supplementary Figure 2: Illustration of six images from patients with proliferative diabetic retinopathy (PDR), with zooms illustrating the local relationship between the non-perfusion areas (NPAs) and the presence of new vessels or intraretinal microvascular abnormalities (IRMA).

SUPPLEMENTARY TABLES

Parameters	Statistical test
**Age**	Rho = 0.079, p = 0.58
**Gender: Male *vs.* Female**	10.87 ± 13.02% *vs.* 16.92 ± 12.49%, p = 0.018*
**Diabetes type: type 1 *vs.* type 2**	12.06 ± 8.41% vs 13.42 ± 15.42%, p = 0.69
**Diabetes control: HbA1c level**	Rho = -0.05, p = 0.74
**Treatment type: oral *vs.* insulin**	10.97 ± 13.98% vs 11.95 ± 7.91%, p = 0.15
**Hypertension**	8.67 ± 6.67 *vs.* 12.22 ± 11.28%, p = 0.44
**BCVA in LogMAR**	Rho = 0.281, p = 0.051

Supplementary Table 1: Correlation between the non-perfusion index and the patients' characteristics.

Assessment of the association between the non-perfusion index (NPI, non-perfusion area percentage) and several demographics and clinical characteristics.

Categorical variables were compared using a Student's t test (when the group size was sufficient) or a non-parametric Wilcoxon's test (when the group size was insufficient). Quantitative variables were compared using a Pearson's test (if normally distributed) or a Spearman correlation test (if not normally distributed). Only the gender was significantly associated with the NPI, probably due to an uneven distribution of the gender between the PDR and NPDR groups (65% of women in the PDR group compared to 38% of men).

* Statistically significant p-value.

Abbreviations: HbA1c: hemoglobin A1c; BCVA: best-corrected visual acuity; LogMAR: logarithm of the Minimal Angle of Resolution.

Sectors	Cutoff	Se	Sp	PPV	NPV	AUC
**Whole image**	12.69	0.625	0.889	0.824	0.706	**0.770**
**ST**	15.79	0.542	1.000	1.000	0.692	**0.784**
**IT**	10.41	0.667	0.778	0.714	0.700	**0.773**
SN	12.92	0.625	0.815	0.737	0.688	0.685
IN	14.01	0.667	0.704	0.652	0.679	0.667
C1	0.23	0.333	0.963	0.875	0.605	0.647
C2	1.44	0.417	0.889	0.750	0.615	0.591
C3	4.66	0.792	0.593	0.621	0.727	0.705
**C4**	15.22	0.792	0.741	0.720	0.769	**0.792**
**ST4**	13.09	0.792	0.778	0.750	0.778	**0.799**
ST3	14.42	0.458	1.000	1.000	0.659	0.702
ST2	0.47	0.542	0.667	0.571	0.600	0.562
ST1	0.01	0.333	1.000	1.000	0.614	0.667
IT4	16.82	0.750	0.778	0.739	0.750	0.746
IT3	17.25	0.458	0.926	0.833	0.641	0.716
IT2	2.16	0.417	0.926	0.818	0.625	0.647
IT1	0.45	0.292	0.963	0.857	0.591	0.625

SN4	17.57	0.667	0.815	0.750	0.710	0.690
SN3	0.36	0.875	0.296	0.513	0.667	0.525
IN4	20.15	0.667	0.704	0.652	0.679	0.710
IN3	13.43	0.292	0.926	0.750	0.581	0.538

Supplementary Table 2: Diagnostic performances of the non-perfusion index in different sectors of the OCTA image.

Results of the analyzes of the diagnostic performances of the non-perfusion index (NPI) measured on the whole image and in the different sectors for the diagnosis of proliferative diabetic retinopathy severity stage.

The top-five area under the curve (AUC) values (in bold) were 0.770 on the whole image, 0.773 in the infero-temporal quadrant, 0.784 in the supero-temporal quadrant, 0.792 in the C4 sector (corresponding to the periphery of the OCTA image outside the most peripheral circle), and 0.799 in the supero-temporal sector of C4.

All sectors are defined and shown in Figure 1B.

Cutoff: optimal (Youden Index) cutoff of the NPI (percentage).

Abbreviations: Se: sensitivity; Sp: specificity; PPV: positive predictive value; NPV: negative predictive value; AUC: area under the receiver operating characteristic (ROC) curve; IN: infero-nasal quadrant; SN: supero-nasal quadrant; IT: infero-temporal quadrant; ST: supero-temporal quadrant; Numbers associated with quadrant abbreviation: 1 = 1st circle, centered on the fovea with a radius of half the optic disc-fovea distance (DFD); 2 = 2nd circle minus 1st circle, centered on the fovea with a radius of 1 DFD; 3 = 3rd circle minus 2nd circle, centered on the fovea with a radius of 2 DFD; 4 = 4th circle, corresponding to the rest of the image (whole image minus 3rd circle); Example: IN3 corresponds to the infero-nasal quadrant intersected with the 3rd circle.

Wilcoxon test, p-value		Kruskal-Wallis test, p-value
**NVE outside *vs.* NVE inside the image field**	0.0499	0.0056
**NVE outside the image field *vs.* NVD**	0.0286	
**NVE inside the image field *vs.* NVD**	0.0074	

Supplementary Table 3: Non-perfusion distribution in the different subgroups of proliferative diabetic retinopathy eyes.

In the PDR group, statistical comparison of the overall NPI between eyes with NVE outside the image field, NVE inside the image field, or NVD. An overall trend statistical analysis was performed using a Kruskal-Wallis test and 2-by-2 comparisons using a Wilcoxon test. In the proliferative diabetic retinopathy (PDR) group, statistical comparison of the overall non-perfusion index (NPI, non-perfusion area percentage) between eyes with new vessels elsewhere (NVE) outside the image field, NVE inside the image field, or new vessels in the optic disc (NVD). There was a significant trend towards an increase in NPI between eyes with NVE outside the image field (6.15 ± 4.91%, with 3 out of 4 cases less than 6.65%), eyes with NVE inside the image field (15.33 ± 7.73%), and eyes with NVD (46.13 ± 21.7%). The 2-by-2 comparisons of the 3 groups were all statistically significant.

Abbreviations: PDR: proliferative diabetic retinopathy; NVE: new vessels elsewhere; NVD: new vessels in the optic disc.

ST4	ST3	ST2	ST1	IT4	IT3	IT2	IT1	SN4	SN3	IN4	IN3
19.61	27.45	7.84	1.96	13.73	27.45	9.80	1.96	27.45	11.76	23.53	17.65

Supplemental Table 4: Percentage of proliferation in different sectors of the OCTA image.

Location of new vessels in the different retinal sectors in eyes with proliferative diabetic retinopathy. For each sector, the percentage of eyes with visible new vessels is indicated.

Abbreviations: IN: infero-nasal quadrant; SN: supero-nasal quadrant; IT: infero-temporal quadrant; ST: supero-temporal quadrant; Numbers associated with quadrant abbreviation: 1 = 1st circle, centered on the fovea with a radius of half the optic disc-fovea distance (DFD); 2 = 2nd circle minus 1st circle, centered on the fovea with a radius of 1 DFD; 3 = 3rd circle minus 2nd circle, centered on the fovea with a radius of 2 DFD; 4 = 4th circle, corresponding to the rest of the image (whole image minus 3rd circle); Example: IN3 corresponds to the infero-nasal quadrant intersected with the 3rd circle.
